# Practical Notes on Cutaneous Subjects

**Published:** 1874-03-15

**Authors:** Tilbury Fox


### PRACTICAL NOTES ON CUTANEOUS SUBJECTS.—SUSPECTED RINGWORM (SCURVY HEAD).

BY TILBURY FOX, M.D., F.R.C.P.

*From the London Lancet, Feb.*, 1874.

THE practitioner is very often puzzled to make a diagnosis in cases of suspected ringworm. Cases, especially in schools, are brought to him which exhibit here and there—or it may be only in one small spot on the scalp—"scurfy"-looking places, without, apparently, any diseased hairs, and he is asked, "Is it ringworm?" Without the microscope, it is difficult to decide the question; and I would venture to say that, under such circumstances, the observer can only blame himself if he falls into error by neglecting the use of the microscope, which will readily reveal, in all cases, whether or no ringworm is present, by the appearance presented by the scales which can be scraped away from the suspected patch. The scales will always be found to have little bits of diseased hairs entangled in them where ringworm is present, and which diseased hairs are not perhaps visible to the naked eye. The accurate diagnosis of these cases is very important where schools are concerned; and a mistake in not recognizing the nature of these "scurvy spots" may lead to the silent but wholesale propagation of the disease among the healthy. The following case affords an illustration of what I mean:

CASE.—I had been prescribing for one or two children in a certain ladies' school, at different periods during two years, for ringworm of the body and head. When the mistress thought that all ringworm had vanished from amongst her pupils, she, having taken every possible means to detect at the earliest moment the faintest trace of mischief in her pupils' heads, in order to prevent the spread of the disease in her school, sent me her little daughter, aged six years, that I might look at a tiny suspicious-looking spot on the crown of the head. This spot turned out to be ringworm, and I destroyed the disease at once by iodine paint. The next day the niece (aged thirteen) of the school-mistress was sent to me for examination, and I learnt that two years ago the scalp of this child was noticed to be "slightly scurvy" in one or two patches here and there over the scalp. The hair thinned out slightly, but the place was not bared of hair, nor was it red. The disease "did not look like ringworm; if it had," the aunt remarked to me, "of course advice would have been sought." The child had been treated with a "little ointment" now and then; and a medical man saw her, but did not think it ringworm. The appearance of the disease in the mistress' young child induced that lady to send the niece to me, lest the "scurfy" disease from which she had been suffering might in reality be ringworm. When I examined the head of the niece, there were one or two irregular-shaped spots, the size of a shilling or so, covered over with fine micaceous scales, not devoid of hairs. The hair looked a little thin, but not more so than is commonly seen in slight cases of seborrhœa, nor did the hairs come out too easily or break off; and on a superficial glance there was no appearance of short broken-off hairs, as in ordinary ringworm. On using a magnifying glass, however, and searching over the diseased areas, certain dark-looking portions of hair-shafts came into view, and these did not run in a natural direction, but were out of the line of the normal hairs, and they were, moreover, in some cases twisted and about three or four lines in length. They were concealed in great part by the healthy hairs. There were, perhaps, five or six in each patch of disease. They turned out to be brittle, and portions came away easily when pulled at. Under the microscope the hairs exhibited the ordinary appearance of hairs invaded by the fungus of tinea tonsurans and fungus of luxuriant growth.

*Remarks.*—The above case illustrates a not uncommon occurrence—viz.: the non-detection of the nature of slightly-developed ringworm of the scalp (tinea tonsurans.) Cases of ringworm may present the same characters as those exhibited by the example under notice from the outset and during their whole course; but these characters may be assumed when the disease has become chronic and is supposed to be well, for ringworm leaves behind, in many cases, a surface that gives off for a while furfuraceous desquamations. The diseased patches may be small—the size of a split-pea—or the area of the disease may be larger. In either case there is apparently a little scurfiness, and the hair is somewhat thinned, and that is all, save an occasional suppurating hair-follicle in the center of the scurfy spot. But if the scales be scraped away, here and there a bit of opaque-looking hair may be seen attached to or projecting from them, and these bits of hair will be found to be crammed full of spores. Further, in all these cases, here and there a dark stub or two, or one or more broken-off hairs, will be detected over the scurfy surface, and afford a certain indication that the disease is parasitic. Very often, as before observed, the condition referred to occurs in a case of ringworm apparently well, and the solitary or few diseased hairs constitute so many spore manufactories to spread the disease, if no parasiticide remedies are used.

The treatment of these cases consists in very carefully getting away every particle of scaliness, and fully epilating the scurfy area, and applying any simple parasiticide until the hair grows healthily again; epilation being repeated to get rid of all short, dull, and opaque-looking hairs.

